# Ultra-Sensitive Aptamer-Based Diagnostic Systems for Rapid Detection of All SARS-CoV-2 Variants

**DOI:** 10.3390/ijms26020745

**Published:** 2025-01-16

**Authors:** Sang Won Kim, Min Jung Han, Md Shafiqur Rahman, Heesun Kim, Jung Eun Noh, Myoung Kyu Lee, Meehyein Kim, Jie-Oh Lee, Sung Key Jang

**Affiliations:** 1 Department of Life Sciences, POSTECH Biotech Center, Pohang University of Science and Technology, 77 Cheongam-ro, Nam-gu, Pohang-si 37673, Republic of Korea; ksw5758@postech.ac.kr (S.W.K.);; 2 Division of Integrative Bioscience & Biotechnology, POSTECH Biotech Center, Pohang University of Science and Technology, 77 Cheongam-ro, Nam-gu, Pohang-si 37673, Republic of Korea; 3 Infectious Diseases Therapeutic Research Center, Korea Research Institute of Chemical Technology (KRICT), 141 Gajeong-ro, Yuseong, Daejeon 34114, Republic of Korea

**Keywords:** SELEX, aptamer, SARS-CoV-2, diagnosis, LFA, PCR

## Abstract

The emergence of numerous SARS-CoV-2 variants, characterized by mutations in the viral RNA genome and target proteins, has presented challenges for accurate COVID-19 diagnosis. To address this, we developed universal aptamer probes capable of binding to the spike proteins of SARS-CoV-2 variants, including highly mutated strains like Omicron. These aptamers were identified through protein-based SELEX using spike proteins from three key variants (D614G-substituted Wuhan-Hu-1, Delta, and Omicron) and virus-based SELEX, known as viro-SELEX. Leveraging these universal aptamers, we created a highly sensitive lateral flow assay (LFA) and an ultra-sensitive molecular diagnostic platform that integrates a novel rapid PCR technique, enabling fast and reliable detection across all SARS-CoV-2 variants.

## 1. Introduction

Since its initial identification in Wuhan, China, in 2019, the coronavirus SARS-CoV-2 has rapidly spread worldwide, resulting in the COVID-19 pandemic [1]. As of September 2024, there have been over 776 million confirmed cases of COVID-19 worldwide, with more than 7.0 million deaths reported [2]. Efforts to control the pandemic have centered on accurate diagnostics, vaccine development, and effective treatment of patients. Despite these efforts, SARS-CoV-2 continues to circulate, evolving into new variants that can evade immunity from prior infections or vaccinations, with Omicron variants now being the most prevalent [3]. SARS-CoV-2 employs its spike protein to bind to the human angiotensin-converting enzyme 2 (ACE2) receptor, facilitating viral entry [4]. The S1 subunit of the spike protein contains the receptor-binding domain (RBD), which interacts with ACE2 and serves as a primary target for neutralizing agents such as antibodies [5]. Mutations in the spike protein have allowed the virus to escape neutralization, contributing to the emergence of variants like Alpha, Beta, Gamma, Delta, and Omicron [3]. The ongoing emergence of new variants presents significant challenges in the development of diagnostic systems. Conventional diagnostic methods primarily target viral genomic materials, such as RNA or DNA in molecular diagnostics [6,7], or viral proteins encoded by the virus in immunological diagnostics [8,9]. However, the rapid mutation of these targets necessitates the identification of highly conserved regions in the viral genome for molecular diagnostics and conserved B-cell epitopes for treatment and immunodiagnostics [10]. Predicting which viral sequences will remain conserved, particularly in future dominant variants, is highly challenging [11]. Likewise, developing monoclonal antibodies that target conserved epitopes demands considerable effort, including the production of numerous antibodies and evaluating their binding affinities across various viral variants [12,13].

Accurate and rapid diagnosis is crucial for effective public health response, particularly in the face of rapidly spreading variants. The gold standard for SARS-CoV-2 detection was Reverse Transcription–quantitative Polymerase Chain Reaction (RT-qPCR), which offers high sensitivity. However, RT-qPCR is associated with high costs, requires skilled technicians, and is time-consuming [14]. As a result, LFAs have emerged as a valuable alternative diagnostic tool. LFAs can detect antigens within 20 min and are more cost-effective than RT-qPCR, although they generally have lower sensitivity [15].

Antigen detection has traditionally relied on antibodies; however, aptamers offer a promising alternative. Aptamers are single-stranded oligonucleotides capable of binding specific targets, including proteins and small molecules [16]. Aptamers are generated through the Systematic Evolution of Ligands by Exponential Enrichment (SELEX) process, which selects candidates from a diverse library using both positive and negative selection [17]. Various SELEX techniques have been developed to enhance the selection process, tailored to the target material and intended application [18,19,20,21,22,23,24].

Aptamers present several advantages over antibodies, including lower batch-to-batch variability, ease of chemical modification, reduced cost, and faster production [25]. Unlike antibodies, which are produced in vivo by injecting antigens into animals, aptamers are selected in vitro, significantly shortening development timelines [26,27]. While phage display technology allows for in vitro antibody screening, aptamer libraries generally offer greater diversity, enhancing the likelihood of identifying high-affinity candidates [28,29,30]. Additionally, aptamers can be rapidly synthesized through chemical processes without the need of cells, making them especially valuable in time-sensitive situations, such as pandemics [31].

**Figure 1:**
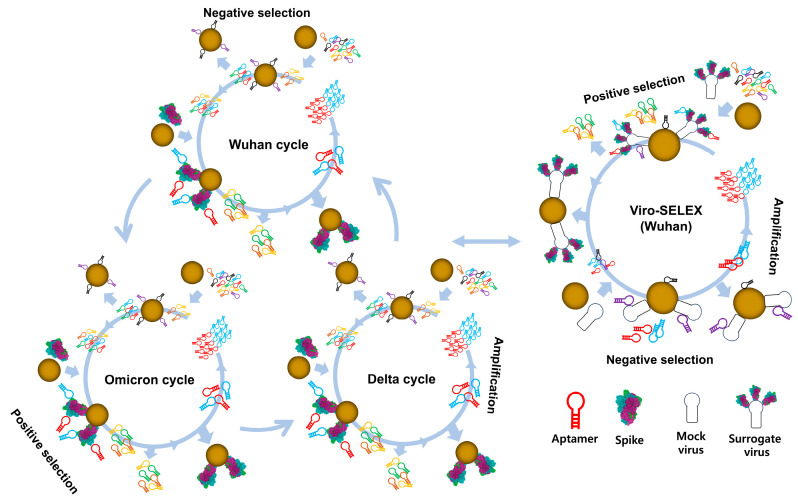
Schematic representation of the juggled viro-SELEX method for consecutive positive selection of aptamers against multiple proteins and a surrogate virus. In contrast to conventional SELEX, which typically targets a single protein, this novel approach incorporates three target proteins and a surrogate virus into the selection process. The method begins with a negative selection step to eliminate nonspecific bead-binding oligonucleotides, followed by consecutive positive selection rounds to enrich oligonucleotides that specifically bind the target proteins or viruses displaying the target protein. The enriched oligonucleotides are then amplified via PCR for subsequent SELEX rounds. The structure of trimeric SARS-CoV-2 spike (PDB ID: 6VSB) is depicted in figure [32].

In this study, we report the identification of two aptamers, AM016 and AM086, through a novel juggled viro-SELEX approach (Figure 1). This method employed purified spike proteins from three SARS-CoV-2 variants—Wuhan with the D614G mutation, Delta, and Omicron—along with a surrogate virus displaying the spike protein on its viral envelope during successive positive selection cycles. This approach successfully identified universal aptamers with broad binding capability across multiple SARS-CoV-2 variants. These aptamers bind to the S1 domain and the RBD of the spike protein, respectively, demonstrating sub-nanomolar affinity, even against the Omicron variant. Additionally, we optimized the aptamers and incorporated them into diagnostic platforms, including LFAs for rapid testing, as well as a newly developed two-step qPCR, which improves sensitivity while reducing processing time.

## 2. Results

### 2.1. Generation of Aptamers with High Affinities to the Spike Proteins of SARS-CoV-2 Variants

To generate aptamers with high affinity for multiple SARS-CoV-2 spike protein variants, we utilized the viro-SELEX technique, based on a previously reported protocol [33]. A key innovation in our approach was the use of a modified nucleoside, Nap-dU (5-[N-(1-naphthylmethyl) carboxamide]-2′-deoxyuridine), in place of deoxythymidine. This substitution in the SELEX process, as previously shown [24,34], enabled the development of a broad repertoire of aptamers with high affinity for SARS-CoV-2 spike proteins. The enhanced binding is likely due to the hydrophobic moiety of the modified nucleoside, which interacts with hydrophobic regions of the target proteins [35].

Our selection process involved consecutive rounds of positive selection using three spike protein variants: the Wuhan strain with the D614G mutation, the Delta variant, and the Omicron variant, as depicted in Figure 1. Trimeric spike proteins, incorporating the foldon domain, were produced using a baculovirus expression system in insect cells (Sf9) and subsequently purified via Ni-NTA affinity chromatography and size-exclusion chromatography (Supplementary Figure S1).

This sequential selection strategy enriched aptamers capable of binding to all three spike variants, suggesting that these aptamers target conserved regions of the spike protein. To further enhance specificity, we included an enrichment step using a specialized baculovirus displaying the SARS-CoV-2 spike protein on the virion envelope. This viro-SELEX method enables the exclusion of aptamers that bind to artificial surfaces of purified proteins, including the foldon domain derived from T4 fibritin. This ensures the selection of aptamers that specifically interact with the native form of the spike protein [21]. Detailed conditions for protein binding during the SELEX process are provided in Table S1.

Next-generation sequencing (NGS) was used to analyze the oligonucleotide sequences enriched after 23 rounds of SELEX (Table S2). The most abundant sequence, AM086, and the second most abundant, AM016, were synthesized and further analyzed to assess their binding affinities to the spike proteins. Notably, several sequences similar to AM016 were also enriched (Table S2, sequences 5, 6, 8, 9, 13, and 17).

**Figure 2:**
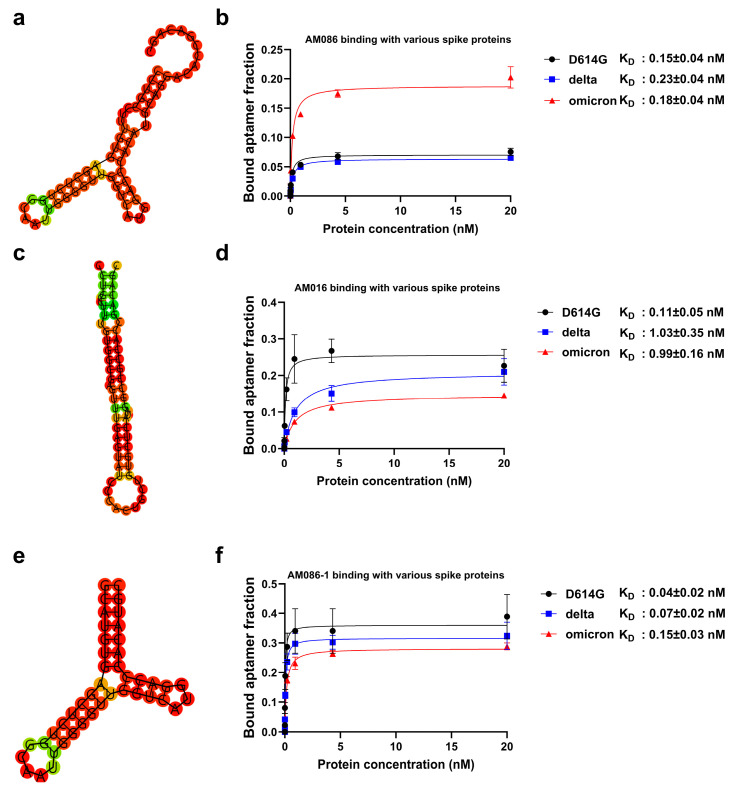
Two-dimensional structure predictions and binding affinities of selected aptamers. The secondary structures of aptamers AM086 (**a**), AM086-1 (**e**), and AM016 (**c**) were predicted using the RNAfold web server (http://rna.tbi.univie.ac.at/cgi-bin/RNAWebSuite/RNAfold.cgi accessed on 16 March 2023) [36,37]. Binding affinities of AM086 (**b**), AM086-1 (**f**), and AM016 (**d**) were assessed using a filter binding assay, as described in the Section 4. AM086 (**b**) exhibits sub-nanomolar K_D_ values for all variants used in the SELEX process. AM016 (**d**) demonstrates sub-nanomolar K_D_ values for the Wuhan (D614G) variant and nanomolar K_D_ values for the Delta and Omicron variants. AM086-1 (**f**), an optimized version of AM086, shows enhanced binding affinity compared to the original aptamer.

Binding affinities of aptamers AM086 and AM016 to the spike proteins of the SARS-CoV-2 variants (Wuhan with D614G mutation, Delta, and Omicron) were determined using filter binding assays [34]. AM086 exhibited K_D_ values ranging from 0.15 nM to 0.23 nM across the variants (Figure 2b), while AM016 showed K_D_ values ranging from 0.11 nM to 1.03 nM (Figure 2d). To optimize AM086, we reduced its size and improved structural stability by incorporating terminal base-pairing and eliminating the bulge structure from the stem. The resulting variant, AM086-1 (Figure 2e), exhibited significantly enhanced binding affinities, with K_D_ values ranging from 0.04 nM to 0.15 nM. Consequently, AM086-1 was incorporated into diagnostic platforms. In contrast, similar optimization strategies applied to AM016 were ineffective, likely due to the intricate stem structure, which may be critical to its binding mechanism (Figure 2c).

Filter binding assays demonstrated that AM086 and AM016 interact with different regions of the spike protein. Specifically, AM086 binds to the RBD within the S1 subunit (Figure S2e), whereas AM016 targets a distinct site in the S1 subunit that is outside the RBD (Figure S2d). This differentiation in binding sites highlights the complementary roles of AM086 and AM016, with AM086 targeting the RBD and AM016 binding to a non-RBD region within the S1 subunit. These distinct binding patterns offer valuable potential for diagnostic and therapeutic strategies.

### 2.2. Development of an LFA System for SARS-CoV-2 Utilizing Aptamers

We developed an LFA system for the detection of SARS-CoV-2, utilizing two aptamers, AM086-1 and AM016. The design of this system follows the methodology outlined in previous studies [38]. In this assay, AM086-1 was employed as the capture probe, while biotinylated AM016 served as the detection probe.

The capture probe AM086-1 was directly immobilized onto the LFA strip, while biotinylated AM016 was conjugated to streptavidin-coated gold nanoparticles (SA-GNP) to enable signal detection. Upon recognition of the target spike protein, a characteristic red line appeared, indicating a positive result. To validate system performance, NH_2_-oligo-d(T) was anchored to the LFA strip, and biotinylated oligo-d(A) conjugated to SA-GNP was added to the sample mixture, ensuring consistent and reliable signal generation. Additionally, a 3′-inverted dT residue was introduced to block 3′ to 5′ exonuclease activity, thereby enhancing the stability of both the capture and detector aptamers [39].

**Figure 3:**
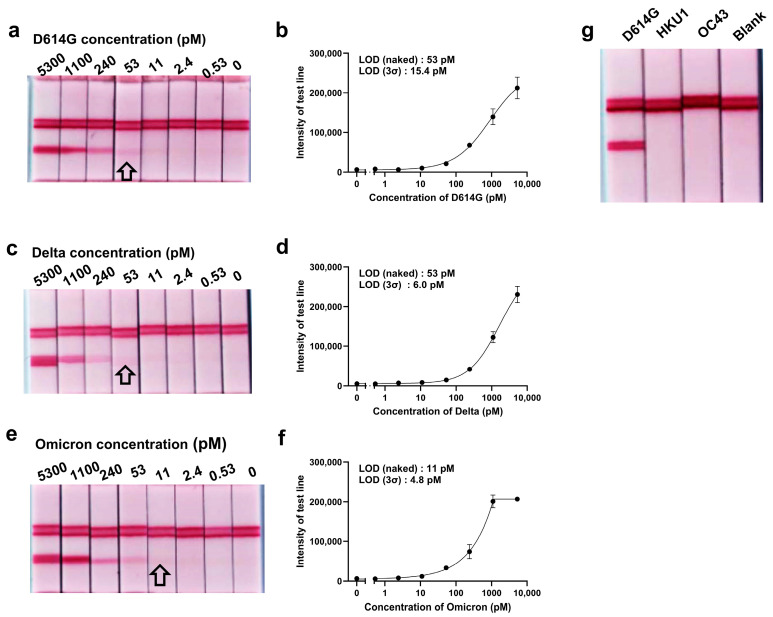
Detection of the SARS-CoV-2 spike protein using an aptamer-based LFA system. The LOD was determined both visually and by measuring band intensity using the Imager 680 system. Each test strip contained 5 pmol of the capture aptamer (thiolated AM086-1) and the detection aptamer (biotin-conjugated AM016). The biotin-conjugated AM016 was coupled to streptavidin-conjugated gold nanoparticles (40 nm) for signal generation. Spike proteins from the Wuhan (D614G) (**a**,**b**), Delta (**c**,**d**), and Omicron (**e**,**f**) variants were applied to the sample pad for detection. (**g**) The specificity of the aptamer-based LFA was assessed using purified spike proteins from Wuhan (D614G), HKU1, and OC43. A positive test line signal was observed exclusively for the Wuhan (D614G) spike protein, confirming the specificity of the LFA system. All arrows in the figure point to the strips corresponding to the LOD measured by the naked eyes.

**Figure 4:**
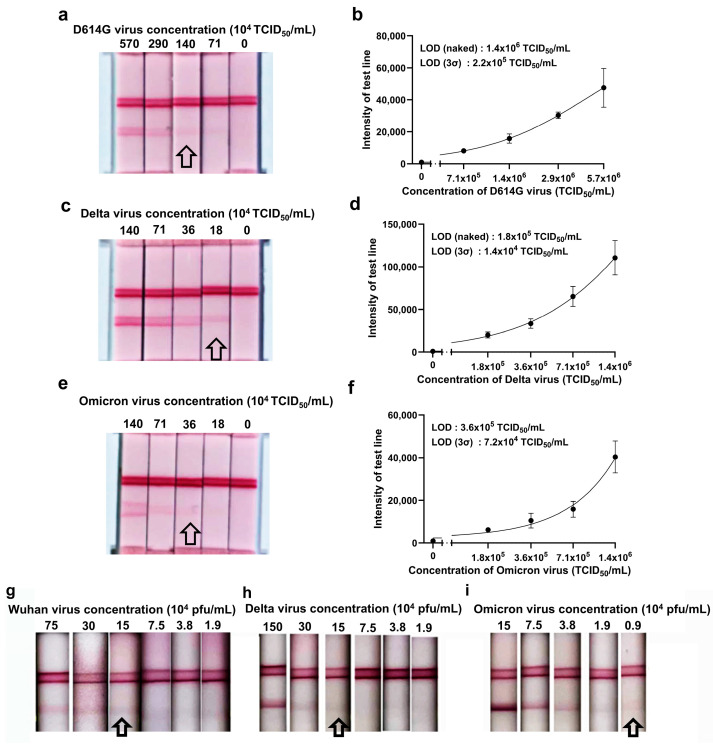
Determination of the LOD for pseudoviruses containing SARS-CoV-2 spike proteins and real SARS-CoV-2 viruses using an aptamer-based LFA system. Pseudoviruses displaying SARS-CoV-2 spike proteins on their envelopes were applied to the sample pad for detection. The LFA was conducted using pseudoviruses from the (**a**,**b**) Wuhan (D614G), (**c**,**d**) Delta, and (**e**,**f**) Omicron variants. The LOD was determined both visually and by quantifying band intensity using the Imager 680 system. Additionally, the aptamer-based LFA system was tested for detecting live SARS-CoV-2 viruses, including the (**g**) Wuhan (NCCP43328), (**h**) Delta (NCCP43390), and (**i**) Omicron (NCCP43408) variants. All experiments involving live viruses were performed in a BSL-3 laboratory. All arrows in the figure point to the strips corresponding to the LOD measured by the naked eyes.

The sensitivity of the LFA system was assessed using purified spike proteins, pseudoviruses, and live SARS-CoV-2 viruses (Figure 3, Figure 4a–f, Figure S4 and Figure 4g–i, respectively). The system exhibited high sensitivity across three tested variants: the Wuhan strain without the D614G mutation (NCCP 43328), the Delta variant (NCCP 43390), and the Omicron variant (NCCP 43408). For purified spike proteins, the LODs ranged from 11.3 to 52.3 pM when assessed visually and from 5 to 15 pM based on 3-sigma calculations (Figure 3a–f), demonstrating the system’s capacity to detect low concentrations of viral proteins.

For pseudoviruses, the LODs determined visually were 1.4 × 10^6^ TCID_50_/mL for the Wuhan variant, 1.8 × 10^5^ TCID_50_/mL for the Delta variant, and 3.6 × 10^5^ TCID_50_/mL for the Omicron variant (Figure 4a–f). Using the 3-sigma calculation, the LODs improved to 2.2 × 10^5^ TCID_50_/mL for the Wuhan variant, 1.4 × 10^4^ TCID_50_/mL for the Delta variant, and 7.2 × 10^4^ TCID_50_/mL for the Omicron variant (Figure 4b,d,f). For live SARS-CoV-2, the LODs were 1.5 × 10^5^ PFU/mL for both the wild-type Wuhan-like strain and the Delta variant and 0.9 × 10^4^ PFU/mL for the Omicron variant (Figure 4g–i).

To assess the specificity of the LFA system, we tested its response to the spike proteins of two human beta coronaviruses associated with the common cold, OC43 and HKU1, which are distinct from SARS-CoV-2 [40]. The LFA system produced no detectable signal upon exposure to these coronaviruses (Figure 3g), demonstrating its high specificity for the SARS-CoV-2 spike protein without cross-reactivity to other beta coronaviruses.

These results indicate that the aptamer-based LFA system provides both high sensitivity and specificity for detecting SARS-CoV-2 and its variants, making it a viable tool for rapid diagnostic applications.

### 2.3. Development of a Rapid and Ultra-Sensitive Diagnostic System by qPCR of DNA Aptamers

In this study, we developed an ultra-sensitive diagnostic system for SARS-CoV-2 detection by integrating a new quantitative Polymerase Chain Reaction (qPCR) technology with DNA aptamers. This novel approach aims to significantly enhance the sensitivity of LFAs described above. Traditional molecular diagnostic platforms, such as RT-qPCR, detect coronavirus genomic RNA by first converting it into complementary DNA (cDNA) via reverse transcription, followed by PCR amplification. In contrast, our system uses DNA aptamers as templates for PCR amplification, bypassing the need for viral RNA conversion to cDNA. This simplifies the detection process and shortens the time required for diagnosis.

**Figure 5:**
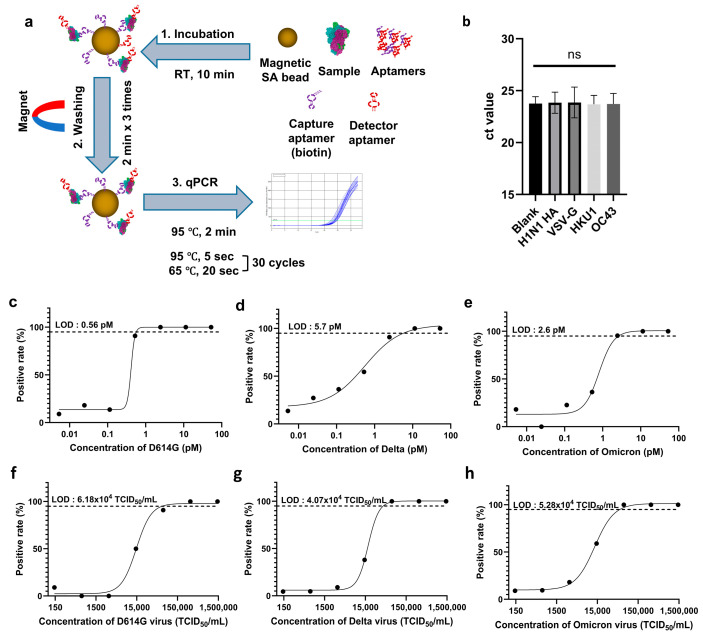
LOD determination for the aptamer-based two-step qPCR diagnostic system. (**a**) A schematic overview of the aptamer-based two-step qPCR system is shown, highlighting rapid sample preparation within 20 min, followed by a two-step qPCR analysis completed in approximately 40 min, resulting in a total diagnostic time of about 1 h. LOD was determined for both protein and pseudovirus targets. (**b**) Specificity of the qPCR method was evaluated using negative control proteins, including hemagglutinin from human influenza virus H1N1 (H1N1 HA), G proteins from vesicular stomatitis virus (VSV-G), and spike proteins from coronaviruses HKU1 and OC43. Across more than 20 replicate tests for each negative control, only non-significant (ns) signals were detected, confirming the method’s specificity. Panels (**c**–**e**) display LOD determinations for specific protein variants: (**c**) Wuhan (D614G), (**d**) Delta, and (**e**) Omicron. Similarly, panels (**f**–**h**) show LOD determinations for pseudovirus targets: (**f**) Wuhan (D614G), (**g**) Delta, and (**h**) Omicron. For each variant, proteins and viruses were incubated with a specific pair of aptamers (capture and detection aptamers), and the amount of enriched detection aptamer was quantified via two-step qPCR. Each concentration was tested in over 20 replicates to ensure reproducibility and validation of the results.

Our system employs a dual aptamer-based design, similar to the configuration used in LFAs. Biotinylated aptamer AM016 functions as the capture element, binding to streptavidin-coated magnetic beads, while aptamer AM086-1 serves as the detection probe, with its signal amplified via qPCR. After binding and washing steps, the M086-1 DNA was thermally denatured at 95 °C for 2 min, followed by qPCR amplification over 30 cycles. Each cycle included denaturation at 95 °C for 5 s and annealing/extension at 65 °C for 20 s (Figure 5a). This streamlined two-step qPCR cycle, with shorter phase durations, provides a faster alternative to the conventional three-step process.

The LOD for the system was defined as the concentration at which 95% of samples tested positive. For protein samples, the two-step qPCR-based system demonstrated significantly improved sensitivity compared to the LFA system, achieving an LOD range of 500 fM to 5 pM (Figure 5b–d), whereas the LFA system had an LOD range of 10 to 50 pM. For pseudoviruses, the LOD was approximately 10^4^ TCID_50_/mL (Figure 5e–g). This qPCR system provided a 5- to 10-fold increase in sensitivity compared to the LFA system while also significantly reducing processing time relative to the conventional RT-qPCR method, which includes a reverse transcription phase and a three-step PCR.

Due to the system’s high sensitivity, nonspecific binding between the aptamers and magnetic beads led to signal detection in blank samples, with a Ct value of 23.8 ± 0.7 (Figure S5). The limit of blank (LOB) was calculated as the mean Ct value of the blank plus 1.65 times the standard deviation (SD). Based on this calculation, a Ct value of 22.7 was set as the cut-off for positive detection [41]. Specificity tests were conducted using human beta coronaviruses OC43 and HKU1. The qPCR system showed no detectable signal when exposed to these viruses (Figure 5b), confirming the high specificity of the aptamer-based qPCR system for SARS-CoV-2.

These findings demonstrate that the two-step qPCR method provides a highly sensitive and specific platform for SARS-CoV-2 detection, offering a promising enhancement over existing diagnostic systems.

## 3. Discussion

In this study, we successfully identified aptamers capable of binding to the spike proteins of multiple SARS-CoV-2 variants. After 26 rounds of SELEX, several aptamers with high sequence prevalence were identified through NGS analysis (Table S2). Most of these aptamers exhibited strong binding affinity to the Wuhan variant with the D614G mutation, as well as the Delta and Omicron variants (Figure S2a–c). However, some aptamers showed reduced affinity for the Omicron variant, despite Omicron-specific selection during SELEX. This reduction in affinity may be attributed to structural changes in Omicron, which affect its entry pathway, unlike the structurally similar Wuhan (D614G) and Delta variants [42]. Nonetheless, two top-ranked aptamers retained high binding affinity across all variants, including Omicron (Figure 2a–d and Figure S3a,b).

Conducting SELEX with a single protein can lead to reduced binding efficacy when significant mutations, such as those observed in the Omicron variant, emerge, as demonstrated in previous studies [33]. This challenge is not limited to aptamers; antibodies similarly exhibit decreased effectiveness against evolving viral mutations [43]. Relying on random identification of aptamers or antibodies that remain effective against future variants poses considerable risk. In contrast, our approach—performing SELEX with multiple viral variants—introduces selective pressure to enrich aptamers that bind to conserved regions across various mutations. This method is particularly advantageous in pandemic scenarios like COVID-19, where viral mutations occur rapidly and frequently.

To evaluate the diagnostic potential of the universal aptamers we identified, we incorporated them into an LFA, a widely used platform for rapid diagnostics. At the protein level, the LOD of the LFA was between 10 and 50 pM. Given that a single virus particle contains about 24 spike trimers, allowing for the binding of up to 72 aptamers [44], the detection sensitivity improves when whole viruses are used. In tests with pseudoviruses, the LOD ranged from 10^5^ to 10^6^ TCID_50_/mL, while for live Omicron virus, the LOD was approximately 10^4^ pfu/mL, corresponding to a Ct value of around 20 [45]. These findings indicate that the aptamer-based LFA system can detect viral loads comparable to those of commercially available diagnostic systems using antibody probes. Especially, many commercial kits experience a marked decline in detection sensitivity for samples with Ct values above 24, whereas our system remains competitive and offers potential for further optimization [45,46]. Additionally, some commercial kits (13/44) exhibit similar LODs (greater than 0.5 × 10^4^ TCID_50_/mL) when tested with live viruses [47].

Our LFA system also demonstrated superior sensitivity compared to previously reported aptamer-based LFA systems, which exhibited LODs of 100 pM [48], 300 pM [49], and 680 pM [50]. Furthermore, our system demonstrated no cross-reactivity with other human beta coronaviruses, such as OC43 and HKU1, which are associated with the common cold (Figure 3g).

To enhance sensitivity, we developed a novel two-step qPCR method capable of detecting lower viral loads with minimal reaction time. The two-step qPCR system demonstrated a LOD ranging from 500 fM to 5 pM at the protein level, significantly surpassing the sensitivity of the LFA system. In pseudovirus tests, the two-step qPCR system achieved a LOD of approximately 10^4^ TCID_50_/mL, marking a 5- to 10-fold improvement in sensitivity over the LFA. This indicates that the two-step qPCR can detect viral loads corresponding to Ct values between 23 and 24 in live virus samples [45]. The method was optimized for speed by minimizing both denaturation and reaction times, allowing for diagnosis within 40 min, utilizing a 5-s denaturation and a 20-s annealing/extension step (Figure 5a). Importantly, no significant differences were observed between blank samples and human beta coronaviruses, demonstrating the high specificity of our two-step qPCR diagnostic system (Figure 5b).

While many commercial diagnostic kits target the nucleocapsid (N) protein [47,51], which is approximately 50 times more abundant than the spike protein [52], our spike protein-targeting approach produced results comparable to those of existing diagnostic systems. This underscores the potential of our qPCR method as a viable alternative to antibody-based diagnostics, especially in the context of rapidly evolving viral mutations.

It would have been ideal to evaluate our diagnostic systems using specimens from real patients. However, this was not possible due to the limited availability of such specimens and the lack of a BSL-3 facility at the institution where the primary researchers of this study are based. Despite these constraints, we are confident that the diagnostic systems will perform effectively with real specimens. This confidence is based on the robust design of the aptamer probes, which are protected from nuclease degradation by modifications at their 5′ and 3′ ends. Furthermore, a similar aptamer-based LFA system developed for the diagnosis of the influenza virus has demonstrated reliable performance with a throat swab sample of a healthy individual [21], supporting the potential applicability of our system in practical settings.

Nonetheless, evaluating diagnostic systems with clinical samples remains a critical next step. Such evaluations will allow for the optimization of reaction conditions, the establishment of positivity thresholds, and the overall enhancement of the system’s reliability in real-world applications.

## 4. Materials and Methods

### 4.1. Cloning, Expression, and Purification of SARS-CoV-2 Spike Protein

The SARS-CoV-2 spike protein was prepared according to established protocols [33]. In brief, the spike protein was expressed using a baculovirus system, with a C-terminal T4 fibritin trimerization domain (foldon domain) incorporated to preserve its trimeric structure [53]. To improve protein stability, proline substitutions were made at positions 986 and 987, and the furin cleavage site (RRAR, residues 682–685) was mutated to GSAS to prevent cleavage between the S1 and S2 domains, as previously described [32]. The spike gene was cloned into a p42 expression vector, which included a 6× His-tag and FLAG-tag for purification.

SF9 insect cells (2 × 10^6^ cells/mL) were infected with the recombinant baculovirus encoding the SARS-CoV-2 spike gene and cultured for three days. Following this, the media were harvested, and cells and debris were removed by centrifugation at 6000 rpm for 10 min. The clarified media were passed through a Ni-NTA resin (Roche, Basel, Switzerland) to capture the His-tagged spike proteins. The resin was washed with a buffer containing 20 mM Tris-HCl (pH 8.0), 200 mM NaCl, and 45 mM imidazole. The bound spike proteins were eluted with an elution buffer composed of 20 mM Tris-HCl (pH 8.0), 200 mM NaCl, and 200~300 mM imidazole.

The eluted proteins were concentrated using a 100 kDa molecular weight cutoff centricon (Merck) and further purified by size-exclusion chromatography. The purified spike proteins were then aliquoted and stored at −70 °C for future use (Figure S1).

### 4.2. Surrogate Virus Preparation

Surrogate viruses were prepared according to established protocols [21,33]. In brief, the full-length SARS-CoV-2 spike protein was cloned into a baculovirus expression system. Baculoviruses displaying the spike protein on their envelope were generated by transfecting the recombinant bacmid into SF9 cells. Three days post-infection, the viral particles were collected by centrifugation at 500× *g* for 5 min, followed by clarification using a syringe filter.

For further purification, the surrogate virus particles were subjected to ultracentrifugation at 100,000× *g* for 2 h on a sucrose gradient. The resulting viral pellet was carefully resuspended in cold PBS buffer containing 2.5% glycerol and stored at −70 °C for future use.

### 4.3. Aptamer Generation by Viro-SELEX

In our previous work, we developed viro-SELEX, a technique specifically designed to select aptamers that bind to viral envelope proteins [21,33]. In this study, we optimized the viro-SELEX method to improve the selection of aptamers capable of binding a wider range of SARS-CoV-2 spike protein variants.

To achieve this, we conducted 26 rounds of SELEX, alternating between the spike proteins of SARS-CoV-2 variants, including the Wuhan strain (with the D614G mutation), Delta, and Omicron. In one round, we substituted the purified spike protein with a surrogate virus displaying the Wuhan spike protein on its envelope. This adjustment aimed to eliminate aptamers that bind to artificial surfaces present in purified proteins but absent in the native virion. Specifically, we sought to remove aptamers targeting the foldon domain, which is found only in purified trimeric spike proteins but not in their native structure (see Table S1, Figure 1).

The aptamer library used contained a 40-mer random sequence flanked by two 25-mer fixed sequences at both ends. Thymidines in the sequences were replaced with 5-[N-(1-naphthylmethyl) carboxamide]-2′-deoxyuridines (Nap-dUs) to enhance the binding properties of aptamers.

For each round of SELEX, the aptamer library was refolded by heating at 95 °C for 5 min, followed by cooling to 37 °C at a controlled rate (0.1 °C/s) in SB18T buffer [40 mM HEPES (pH 7.5), 102 mM NaCl, 5 mM KCl, 5 mM MgCl_2_, and 0.05% Tween-20]. To eliminate nonspecific binders, the refolded library was incubated with 6× His-tagged TALON beads (Invitrogen, Waltham, MA, USA). After magnetic separation, the supernatant was incubated with purified trimeric spike proteins under varying conditions (e.g., different protein concentrations and the use of dextran sulfate as an anionic competitor), as outlined in Table S1.

Target-bound DNAs were eluted with 2 mM NaOH, washed with SB18T buffer, and neutralized with 8 mM HCl. The eluted DNAs were then PCR-amplified using a 5′-primer (5′-ATATATATCGAGCGTCCTGCCTTTG-3′) and a biotin-labeled 3′-primer [5′-(2×)Biotin-TTTTTTTTCTGGGTGGCTGTCGGTG-3′]. After amplification, the DNAs were linked to MyOne Streptavidin C1 beads (Invitrogen), and only the positive strand was eluted with 20 mM NaOH. To synthesize a new positive strand, the beads were incubated with dATP, dCTP, dGTP, Nap-dU, and KOD enzyme at 68 °C for 30 min. The Nap-dU-containing positive strand was then eluted with 20 mM NaOH and neutralized with 80 mM HCl.

The aptamer pool from the 23rd round was selected for sequencing based on its strong target-binding activity. PCR-amplified DNA from this pool was purified using the QIAquick PCR purification kit (QIAGEN, Hilden, Germany), followed by Sanger sequencing (Solgent, Daejeon, Republic of Korea) and next-generation sequencing (NGS) (LAS, Seoul, Republic of Korea). The top-performing aptamers were synthesized by Aptamer Science (Seongnam-si, Republic of Korea) following previously established protocols [33].

### 4.4. Measurement of the K_D_ Values of Aptamers

The dissociation constant (K_D_) between the aptamer and target protein was determined using a filter binding assay, as described previously [34]. Briefly, the 5′ end of the aptamer was radiolabeled with [^32^P]-γ-ATP (PerkinElmer, Waltham, MA, USA) using T4 polynucleotide kinase (Takara Bio Inc., Kusatsu, Japan). The labeled aptamers were then purified using MicroSpin G-50 columns (Cytiva, Marlborough, MA, USA) with centrifugation at 800× *g* for 1 min. The purified aptamers were diluted in SB18T buffer for subsequent binding assays.

The refolded aptamers were incubated with varying concentrations of the target protein at 37 °C for 30 min. After incubation, 1/30 of the reaction mixture was spotted onto a non-charged nylon membrane (QIAGEN). Zorbax resin (Agilent, Santa Clara, CA, USA) was then added to the remaining mixture and incubated for 1 min. The mixture was applied to a 0.45 μm PVDF 96-well filter plate under vacuum. Following an overnight incubation at −20 °C, the radioactivity retained on the membrane was measured using a phosphor imager (Amersham Typhoon 5, Cyvita, Marlborough, MA, USA). K_D_ values were calculated using Prism 10 software (GraphPad, Boston, MA, USA) by fitting the data to a one-site-specific binding model.

### 4.5. Generation of Pseudoviruses

SARS-CoV-2 pseudoviruses were produced using an HIV-based lentiviral system, in which the SARS-CoV-2 spike gene was modified with a 21-amino-acid deletion at the C-terminal region [38]. HEK293T cells were seeded at 25% confluency in poly-L-lysine (PLL)-coated culture plates and incubated overnight. For transfection, a plasmid mixture containing pHAGE-Luc (NR-52516, BEI Resources, Manassas, VA, USA), HDM-HgpM2 (NR-52517, BEI Resources), HDM-tat1b (NR-52518, BEI Resources), pRC-Rev1b (NR-52519, BEI Resources), and pHDM-SARS-CoV-2 (D614G mutant, NR-53765, BEI Resources), as well as Delta or Omicron variants, was prepared using Fugene HD (Promega, Madison, WI, USA) in Opti-MEM media (Gibco, Grand Island, NE, USA) and applied to HEK293T cells. Media changes were performed at 24 and 48 h post-transfection. After 60 h of incubation, virion particles were collected and filtered through a 0.45 μm syringe filter to remove cellular debris.

### 4.6. Calculation of Pseudovirus Titer

To determine the pseudovirus titer, the experiment was performed with slight modifications to established protocols [54,55]. A total of 3 × 10^4^ 293T-ACE2 cells were seeded into each well of a 96-well plate and incubated for 12 h. The cells were then infected with serially diluted pseudovirus stocks and incubated for 48 h to allow for viral replication. After incubation, the culture media were discarded, and 30 μL of passive lysis buffer (PROMEGA) was added to each well, followed by a 5-min incubation. Subsequently, 30 μL of firefly luciferase substrate was added, and luciferase activity, indicative of viral replication, was measured using a Tecan Infinite M300pro plate reader (Tecan, Männedorf, Switzerland), with a 4-s shaking interval before each reading.

The tissue culture infectious dose 50% (TCID_50_) was calculated by determining the positive cutoff value as the mean of the blank controls plus 3.3 times the standard deviation of the blanks (Figure S4) [56].

LOD = (mean_blank_ + 3.3 × SD_blank_) (1)

TCID_50_ values were then determined based on Reed–Muench method [57].

### 4.7. Preparation of LFA Strip

To construct the LFA strip, we employed a previously established method with specific refinements [38]. For the capture aptamer preparation, the thiolated aptamer was treated with DTT, TEA, and TEAA to reduce disulfide bonds, followed by a 1-hour incubation with shaking at 700 rpm in a thermomixer. The aptamer was then precipitated with ethanol, dissolved in 32 μL of SB18T buffer, heated to 95 °C for 5 min, and slowly cooled to 37 °C at a rate of 0.1 °C/s.

Separately, bovine serum albumin (BSA) was conjugated with sulfo-SMCC by incubating for 1 h at 700 rpm. Excess sulfo-SMCC was removed by centrifugation with a 30 kDa centricon at 1800 rpm for 10 min, followed by two washes with 4 mL of SB18T buffer. The capture aptamer and BSA-SMCC were then mixed in a 1:2 molar ratio and incubated for 30 min with shaking at 700 rpm.

A Zeta dispenser machine (Gunpo-si, Republic of Korea) was used to apply lines precisely onto a nitrocellulose membrane sheet, dispensing at 1 μL/cm. The capture aptamer (5 pmol) was applied to the test line, while oligo-d(T) (2 pmol) was applied to the control line. The membrane was incubated at room temperature for three days, after which it was cut into 0.4 cm segments. These segments, along with a sample pad (AP31) and absorption pad (CFSP203000), were assembled onto plastic cassettes to form the LFA strips.

For the preparation of the detector aptamer, the aptamer was heated to 95 °C for 5 min and gradually cooled to 37 °C at a rate of 0.1 °C/s. Streptavidin-conjugated gold nanoparticles (40 nm) were then added to the aptamer solution at a 1.5-fold molar excess, and the mixture was incubated with shaking at 700 rpm for 1 h. To remove unbound aptamers, the mixture was centrifuged at 8000 rpm for 30 min, and the supernatant was discarded. The pellet was washed with washing buffer (1× SB18, 4% sucrose, 0.4% Tween 20) under the same centrifugation conditions. After removing the supernatant, resolution buffer (1× SB18, 4% sucrose, 0.4% Tween 20, 0.5% BSA, and 0.1% NaN_3_) was added to achieve a final detector aptamer concentration of 1 μM.

### 4.8. Determination of Limit of Detection (LOD) for LFA Diagnosis

To determine the LOD for the LFA, the following protocol was used. A solution containing 5 pmol of detector aptamer and 2 pmol of oligo-d(A) was prepared in 70 μL of sample buffer, consisting of 1× SB18 with 4% sucrose, 4% Tween 20, 0.1% NaN_3_, 10 μM DxSO_4_, and 1% Triton X-100. The test sample was diluted in the same buffer to a final volume of 30 μL and combined with the aptamer, d(A) mixture. The resulting mixture was incubated for 5 min at 1400 rpm in a thermomixer, after which 100 μL of the solution was applied to the LFA strip’s sample pad.

After 20 min, the results were assessed both visually and quantitatively using the Amersham Imager 680 to measure the intensity of the test line. Background noise was minimized by applying a rolling ball algorithm with a 5 mm radius for accurate analysis. For protein analysis, the data were fitted to a five-parameter sigmoid model, and the LOD was calculated using the 3-sigma method in Prism 10 software. For pseudovirus analysis, the data were fitted to a hyperbolic model, and the LOD was similarly determined using the 3-sigma method in Prism 10 software.

For assessing non-specific binding, human beta coronaviruses HKU1 and OC43 (purchased from Sino Biological Inc., Beijing, China) were used as negative controls. Additionally, live SARS-CoV-2 virus tests were conducted in a BSL-3 laboratory under the same conditions.

### 4.9. Development of Diagnostic System Using Two-Step Quantitative PCR (qPCR)

For the two-step qPCR diagnosis, the detector aptamer (5 × 10^9^ copies/10 μL) and capture aptamer (10^10^ copies/10 μL) were dissolved in SB18T buffer. The solution was heated to 95 °C for 5 min and then gradually cooled down to 37 °C at a rate of 0.1 °C/s. The sample was diluted in the SB18T, and 40 μL of this diluted sample was mixed with 10 μL of the detector aptamer, 10 μL of the capture aptamer, and 0.7 μL of 0.1 mg/mL streptavidin-coated magnetic beads (C1). This mixture was incubated by shaking for 10 min at 25 °C and 1400 rpm in a thermomixer.

After incubation, the supernatant was carefully removed using a magnetic separator, and 100 μL of SB18T buffer was added for the washing step. The mixture was shaken for 2 min at 25 °C and 1400 rpm in the thermomixer, and the supernatant was removed. This washing process was repeated three times. After the final wash, 50 μL of deionized water was added, followed by shaking for 5 min at 25 °C and 1400 rpm.

For two-step qPCR amplification, 10 μL of the prepared sample was combined with 10 μL of 2× qPCR master mix, which included 2× KOD buffer [210 mM Tris-HCl (pH 7.8), 20 mM KCl, 12 mM (NH_4_)_2_SO_4_, 3 mM MgSO_4_, 0.2 mg/mL BSA, 4% DMSO], 400 nM of forward and reverse primers, 20 mM dNTPs, 10 mM MgCl_2_, 2× SYBR Green, and 0.001 U/μL KOD enzyme. The two-step qPCR was run using the following thermal cycling conditions: 95 °C for 5 s, followed by 65 °C for 20 s for 30 cycles. The primers used were forward (5′ GCATGTGAGCTCTGGC 3′) and reverse (5′ GCATGTGGGTCCATGAC 3′).

To assess non-specific binding, human beta coronaviruses HKU1 and OC43, obtained from Sino Biological Inc., were used.

### 4.10. Determination of LOB and LOD for Two-Step qPCR Diagnosis

To determine the LOB for the two-step qPCR diagnostic system, 24 negative control samples were measured. The LOB was calculated according to its standard definition, using the following formula:

LOB = mean_blank_ ± 1.645 × SD_blank_ (2)

SD_blank_ represents the standard deviation of the blank samples. The cutoff cycle threshold (Ct) value was determined using the upper limit of the LOB, calculated as Equation (3).

LOB = mean_blank_ − 1.645 × SD_blank_ (3)

This equation serves as the threshold for distinguishing between the signal and background noise [41].

To determine the LOD of the two-step qPCR diagnostic system, multiple standard concentrations were tested, with each concentration assayed at least 20 times (20~22 times). Using the predetermined cutoff Ct value, positive and negative samples were distinguished, and the percentage of positive samples at each concentration was calculated. The percentage of positive samples was then plotted against concentration to generate a curve.

The LOD was defined as the concentration at which 95% of the replicates were classified as positive. This curve was fitted using a four-parameter sigmoid model, and the analysis was conducted using Prism 10 software.

## 5. Conclusions

In this study, we identified two aptamers that effectively bind to the spike proteins of multiple SARS-CoV-2 variants, including the Wuhan strain with the D614G mutation, Delta, and Omicron. This demonstrates that our strategy, which involved consecutive positive selection using spike proteins from these three variants combined with viro-SELEX, effectively enriched aptamers targeting conserved regions across these variants. Our results indicate that this approach enhances the potential to isolate aptamers with broad-spectrum binding capabilities, which may prove effective against emerging variants in the future. Such universal aptamers hold significant promise for the development of diagnostic systems capable of addressing rapidly mutating viruses.

Furthermore, we developed an innovative molecular diagnostic system using these universal aptamers, which bind to spike proteins from multiple SARS-CoV-2 variants. By eliminating the reverse transcription step and simplifying the PCR protocol, we significantly reduced the overall diagnostic time without compromising sensitivity.

## Data Availability

Data are included within this article.

## Supplementary Materials

The following supporting information can be downloaded at: https://www.mdpi.com/article/10.3390/ijms26020745/s1.
