# Supplementary material for: Ultra-Sensitive Aptamer-Based Diagnostic Systems for Rapid Detection of All SARS-CoV-2 Variants
Source: Int J Mol Sci. 2025 Jan 16;26(2):745. doi: 10.3390/ijms26020745 (PMC11766214; doi:10.3390/ijms26020745)
Supplement: Supplementary file 1 [file ijms-26-00745-s001.zip › ijms-3367205-supplementary.pdf]

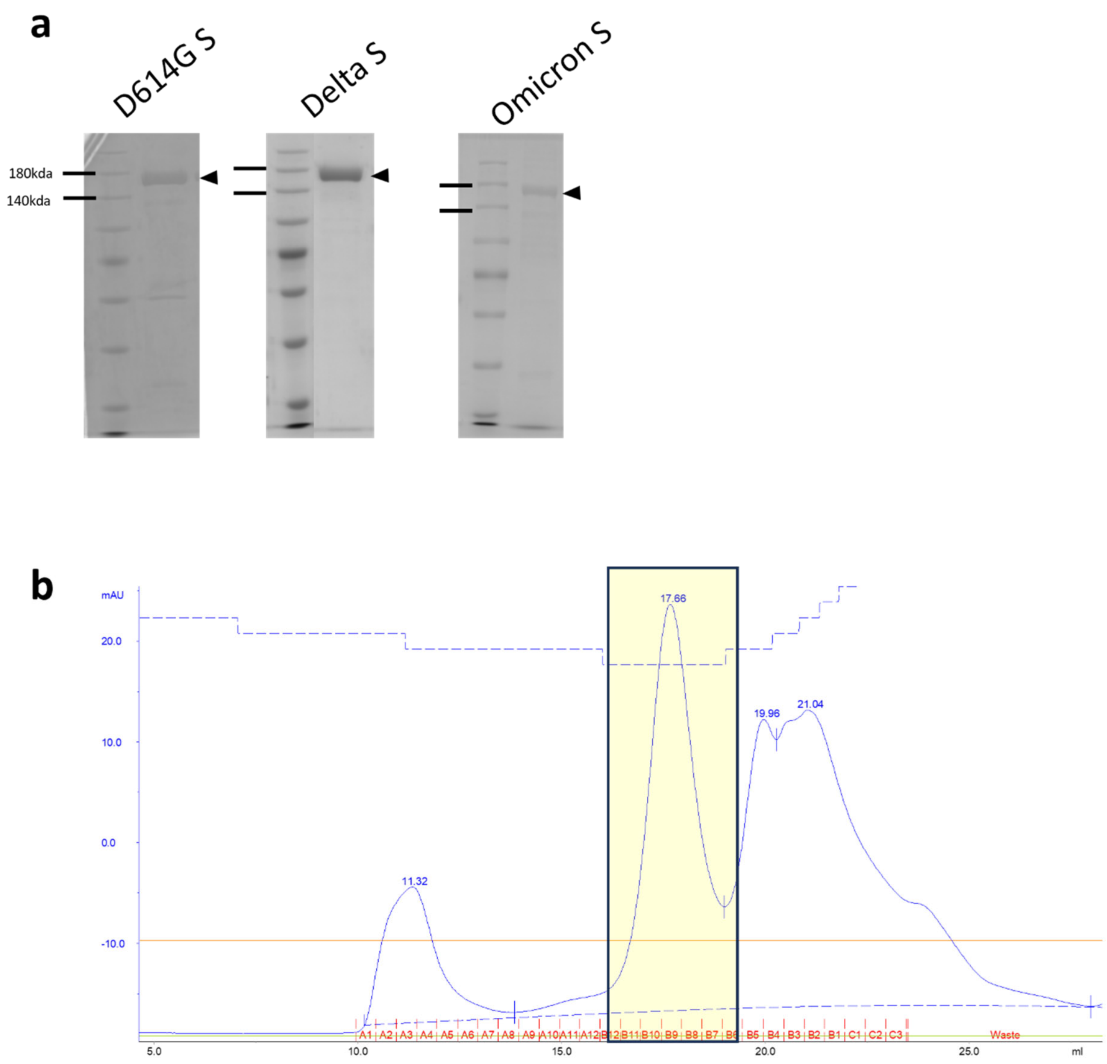


**Supplementary Figure S1.** Preparation of Trimeric Spike Protein. (a) SDS-PAGE analysis of purified His-tagged trimeric spike proteins. Purification was performed using a two-step process: Ni-NTA affinity chromatography followed by gel filtration chromatography. The left, middle, and right panels represent the purification results for the Wuhan (D614G), Delta, and Omicron variants, respectively. (b) Gel filtration chromatography profile. The chromatogram illustrates the purification process, with fractions highlighted in the yellow box indicating those collected for further applications. These fractions were pooled and concentrated for subsequent experiments.

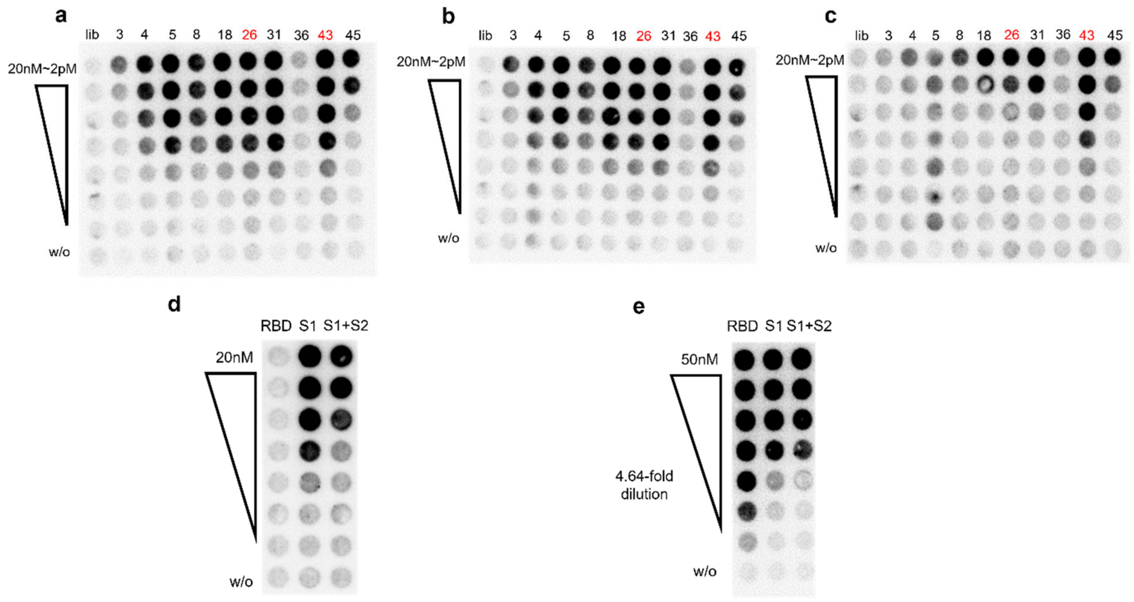


**Supplementary Figure S2.** Binding affinity and binding site determination of aptamers to the target protein using a filter-binding assay. (a–c) Filter-binding assay results for aptamers from the 23rd SELEX pool, tested against spike proteins from the (a) Wuhan (D614G), (b) Delta, and (c) Omicron variants. Aptamers selected for further analysis are highlighted in red, with numbers corresponding to their positions in the Sanger sequencing list. Aptamer #26 is designated as AM016, and aptamer #43 as AM086. (d, e) Binding site determination of aptamers AM016 and AM086 using filter-binding assays with the RBD, S1, and S1+S2 domains of the Wuhan (D614G) spike protein. (d) AM016 binds to the S1 and S1+S2 domains but not the RBD, indicating that it specifically targets the S1 domain outside the RBD. (e) AM086 binds to the RBD, S1, and S1+S2 domains, confirming its specific binding to the RBD.

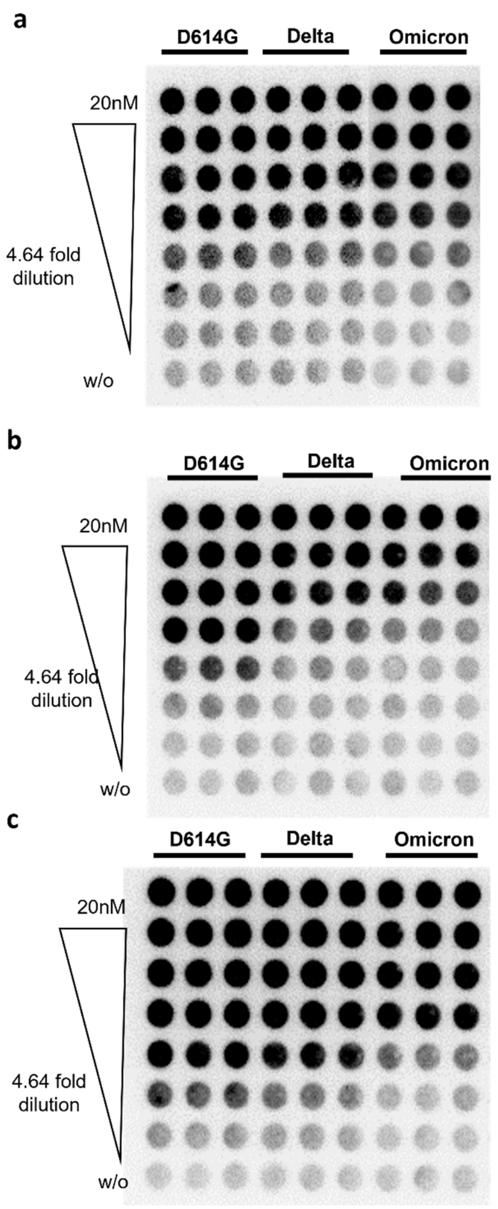


**Supplementary Figure S3.** Determination of $K_D$ values for aptamers shown in Figure 2 using filter-binding assays. (a-c) Triplicate filter-binding assays were performed to determine the $K_D$ values of the aptamers (a) AM086, (b) AM016, and (c) AM086-1 against purified spike proteins from the Wuhan (D614G), Delta, and Omicron variants.

**a**

| | D614G | | | | | Delta | | | | |
|---|---|---|---|---|---|---|---|---|---|---|
| 0.1 | 59,262 | 52,512 | 44,433 | 43,208 | 48,481 | 34,868 | 31,618 | 30,479 | 36,047 | 35,847 |
| 0.01 | 14,263 | 12,034 | 11,747 | 12,606 | 11,800 | 8877 | 7985 | 7884 | 8749 | 9332 |
| 0.001 | 3350 | 3298 | 3707 | 3772 | 3416 | 2928 | 2346 | 2273 | 1970 | 2392 |
| 0.0001 | 1352 | 1430 | 1751 | 1558 | 1466 | 1286 | 1208 | 1041 | 1097 | 938 |
| 0.00001 | 755 | 782 | 830 | 948 | 1013 | 887 | 886 | 789 | 715 | 671 |
| 0.000001 | 479 | 542 | 571 | 614 | 619 | 607 | 559 | 591 | 945 | 463 |
| 1E-07 | 323 | 339 | 398 | 506 | 506 | 438 | 413 | 400 | 368 | 310 |
| 0 | 333 | 338 | 344 | 352 | 373 | 422 | 393 | 349 | 305 | 296 |
| | | LOD | 475.79 | | | | LOD | 475.79 | | |
| | | $TCID_{50}$/mL | 6.81.E+07 | | | | $TCID_{50}$/mL | 2.37.E+07 | | |

**b**

| | Omicron | | | | |
|---|---|---|---|---|---|
| 0.5 | 96,145 | 71,446 | 96,086 | 86,271 | 64,235 |
| 0.05 | 34,745 | 26,720 | 32,636 | 33,374 | 27,027 |
| 0.005 | 9163 | 8134 | 9030 | 7586 | 8524 |
| 0.0005 | 4232 | 4462 | 3448 | 3264 | 3964 |
| 0.00005 | 2825 | 2486 | 2243 | 1888 | 1778 |
| 0.000005 | 1810 | 1705 | 1476 | 1330 | 1313 |
| 5E-07 | 1435 | 1198 | 1139 | 1047 | 1003 |
| 0 | 1140 | 1036 | 1028 | 983 | 1008 |
| | | LOD | 1304.17 | | |
| | | $TCID_{50}$/mL | 8.43.E+06 | | |

**Supplementary Figure S4.** Calculation of $TCID_{50}$ for pseudoviruses. (a, b) Pseudovirus titers were determined through luciferase activity measurements. The cutoff value for luciferase activity was defined using data from negative control wells, and wells were classified as positive or negative based on this threshold. Positive wells are highlighted in green. The $TCID_{50}$/mL was calculated following the standard $TCID_{50}$ methodology [1].

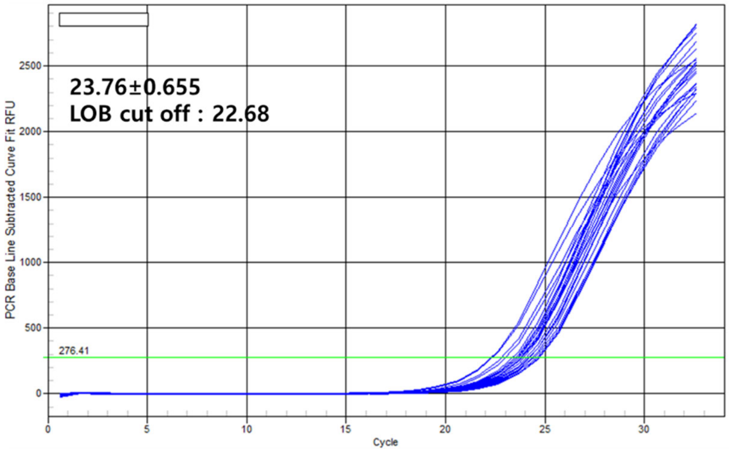


**Supplementary Figure S5.** Determination of the Limit of Blank (LOB) for the aptamer-based qPCR diagnostic system. The cutoff value for the assay was determined from 24 repeated blank tests. The LOB was defined and applied as the threshold to differentiate between positive and negative results.

**Supplementary Table S1.** Reaction conditions for juggled viro-SELEX. In each SELEX round, target proteins were alternated for positive-positive selections using different variants. The amount of target protein was progressively reduced, while the concentration of negatively charged $DxSO_4$ was gradually increased to enrich aptamers with higher binding affinity. $DxSO_4$ was included to minimize non-specific DNA binding during the SELEX process.

| Round | Target molecules (pmole) | Binding condition | Kinetic challenge |
|---|---|---|---|
| 1 | Delta S protein (50) | 1 hour | X |
| 2 | Omicron S protein (10) | 30 minutes | X |
| 3 | D614G S protein (5) | 15 minutes | X |
| 4 | Delta S protein (1) | 15minutes + 0.1 µM $DxSO_4$ | X |
| 5 | Omicron S protein (1) | 15minutes + 0.1 µM $DxSO_4$ | X |
| 6 | D614G S protein (1) | 15minutes + 0.1 µM $DxSO_4$ | X |
| 7 | D614G S protein (1) | 15minutes + 0.1 µM $DxSO_4$ | X |
| 8 | Delta S protein (0.3) | 15minutes + 1 µM $DxSO_4$ | X |
| 9 | Omicron S protein (0.3) | 15minutes + 1 µM $DxSO_4$ | 30 minutes + 10 µM $DxSO_4$ |
| 10 | D614G S protein (0.3) | 15minutes + 1 µM $DxSO_4$ | 30 minutes + 100 µM $DxSO_4$ |
| 11 | Delta S protein (0.1) | 15minutes + 1 µM $DxSO_4$ | 30 minutes + 100 µM $DxSO_4$ |
| 12 | Omicron S protein (0.1) | 15minutes + 10 µM $DxSO_4$ | 30 minutes + 100 µM $DxSO_4$ |
| 13 | D614G S protein (0.1) | 15minutes + 10 µM $DxSO_4$ | 30 minutes + 1 mM $DxSO_4$ |
| 14 | Baculovrius | 15minutes + 10 µM $DxSO_4$ | x |
| 15 | Delta S protein (0.03) | 15minutes + 10 µM $DxSO_4$ | 30 minutes + 1 mM $DxSO_4$ |
| 16 | Omicron S protein (0.03) | 15minutes + 10 µM $DxSO_4$ | 30 minutes + 1 mM $DxSO_4$ |
| 17 | D614G S protein (0.03) | 15minutes + 10 µM $DxSO_4$ | 30 minutes + 1 mM $DxSO_4$ |
| 18 | Delta S protein (0.03) | 15minutes + 10 µM $DxSO_4$ | 30 minutes + 1 mM $DxSO_4$ |
| 19 | Omicron S protein (0.01) | 15minutes + 10 µM $DxSO_4$ | 30 minutes + 1 mM $DxSO_4$ |
| 20 | D614G S protein (0.01) | 15minutes + 10 µM $DxSO_4$ | 30 minutes + 1 mM $DxSO_4$ |
| 21 | Delta S protein (0.003) | 15minutes + 10 µM $DxSO_4$ | 30 minutes + 1 mM $DxSO_4$ |
| 22 | Omicron S protein (0.003) | 15minutes + 10 µM $DxSO_4$ | 30 minutes + 1 mM $DxSO_4$ |
| 23 | D614G S protein (0.003) | 15minutes + 10 µM $DxSO_4$ | 30 minutes + 1 mM $DxSO_4$ |
| 24 | Delta S protein (0.001) | 15minutes + 10 µM $DxSO_4$ | 30 minutes + 1 mM $DxSO_4$ |
| 25 | Omicron S protein (0.001) | 15minutes + 10 µM $DxSO_4$ | 30 minutes + 1 mM $DxSO_4$ |
| 26 | D614G S protein (0.001) | 15minutes + 10 µM $DxSO_4$ | 30 minutes + 1 mM $DxSO_4$ |

**Supplementary Table S2.** NGS analysis of DNA sequences from the 23rd round of SELEX. DNA sequences obtained through next-generation sequencing (NGS) of the 23rd SELEX pool was analyzed. The table lists DNA sequences that occurred more than 10,000 times in the sequencing data.

| RANK | Aptamer Sequence | Count |
|---|---|---|
| **1 (=AM086)** | TGAGCTCTGGCAATTGGGGTTGGTCATGGACCCACATGCAGGA | 94432 |
| **2 (≒AM016)** | CTCTCAGTTTGCATATCCCACTGCTGCTGCTCATATGGCAAA | 66745 |
| 3 | CTGACGGCGTTTGGGTATACAACTGCGTCACTAGCTCATAG | 60422 |
| 4 | TTTGCGTATCCAACTGCTTCCAGCTCATACGTATGGAC | 57190 |
| 5 **(≒AM016)** | GAACAAACGTTTGAGTATAACACTGCCTCCTGCTCATAGTG | 52506 |
| 6 **(≒AM016)** | TGGGAAGTTTGAGTATCCCACTGCTGTGCTCATAGACATCC | 36026 |
| 7 | TCGGTAAGGGAACTAGTGTATGTCCCTGAGTTAAGGATAT | 35261 |
| 8 **(≒AM016)** | AGGTGAGTTTAAGTATCCCACTGCTGTGCTTATAGACAGT | 33268 |
| 9 **(≒AM016)** | AGGTGAGTTTAAGTATCCCACTGCTGTGCTTATAGGCCAT | 30455 |
| 10 | CCTTCTAGTGGTGTCAAACTGTTCCAAAGGACAGGGACA | 23755 |
| 11 | TCGGTAAGGGAACTAGTGTATGTCCCTAAGTTAAGGATAT | 23640 |
| 12 | AACGTTTGCGTATCACACTGGCTACTGCCTCATACGAAGA | 23020 |
| 13 **(≒AM016)** | AGGTGAGTTTAAGTATCCCACTGCTGTGCTTATAGGCCGT | 22848 |
| 14 | ACTGACGGCGTTTGGGTATACAACTGCGTCACTAGCTCATAG | 12904 |
| 15 | CTGTCGGCGTTTGGGTATACAACTGCGTCACTAGCTCATAG | 12408 |
| 16 | GCCGTTTGCGTATCACACTGGCTACTGCCTCATACGAGGA | 12187 |
| 17 **(≒AM016)** | TGAGGAGTTTGAGTATCCCACTGCTGTGCTCATAGGCCCT | 12112 |
| 18 | AACGTTTGCGTATAACACTGGCTGCTGCCTCATACGAAGG | 11061 |

# References


1. Reed, L.J. and H. Muench, *A SIMPLE METHOD OF ESTIMATING FIFTY PER CENT ENDPOINTS.* American Journal of Epidemiology, 1938. **27**: p. 493-497.
